# Immunomodulatory Role of Adipose-Derived Stem Cells on Equine Endometriosis

**DOI:** 10.1155/2015/141485

**Published:** 2015-06-09

**Authors:** Maria Elena Falomo, Letizia Ferroni, Ilaria Tocco, Chiara Gardin, Barbara Zavan

**Affiliations:** ^1^Department of Veterinary Clinical Sciences, Viale dell'Università 16, 35100 Padua, Italy; ^2^Department of Biomedical Sciences, University of Padua, Via G. Colombo 3, 35100 Padua, Italy; ^3^Department of Neurosciences, University of Padua, Via Giustiniani 2, 35100 Padua, Italy

## Abstract

Endometriosis is a degenerative process due to a chronic inflammatory damage leading to extracellular matrix components deposition and glandular fibrosis. It is known that mesenchymal stem cells secrete a wide range of bioactive molecules, some of them modulating the immune inflammatory response, and others providing regeneration and remodeling of injured tissue. We have performed *in vitro* experiments in order to analyze the capability of allogenic equine adipose-derived stem cells (ADSCs) to infiltrate mares' endometrial tissues and to stimulate the expression of cytokines and metallopeptidases. Differences in the biologic response to the exposure to ADSCs between pathological and healthy endometrial tissue have been identified. These results could challenge researchers to progress forward with future studies for the development of a biological therapy with a possible application in translational medicine.

## 1. Introduction

Mesenchymal stem cells (MSCs) are nowadays isolated from many different tissues, including bone marrow, umbilical cord blood and tissue, and adipose tissue, which constitute the most promising alternative for research and clinical purposes due to the easy accessibility. MSCs secrete a wide range of bioactive molecules, some of them modulating the immune inflammatory response, others providing regeneration and remodeling of injured tissue, and finally mitogens acting on lymphatic progenitors differentiation [1–10]. Thus, MSCs gained progressively higher attention in consideration of their bioactive features and started to be vastly used in research to test the effects of their multifactor dispensaries on clinical settings.

Endometriosis is a multifactorial degenerative process of uterine glands and surrounding stroma, and it is considered a major cause of equine infertility [11, 12]. Endometrial tissue degeneration is due to a chronic inflammatory damage leading to extracellular matrix (ECM) components deposition and glandular fibrosis. In mares, the disease seems to be age-related, although a correlation with hormonal changes has not been demonstrated [13–15]. Etiology is still only partially clear, as studies show differences in mares' immune inflammatory response and thus susceptibility of the development of endometriosis [16–18]. Hypothesis has been made that differences in the expression rate of pro- and anti-inflammatory gene products in chronic inflammation are implied [17–19], involving especially ECM degrading-endopeptidases known as metallopeptidases (MMPs) and their inhibitors (TIMPs) [20, 21].

We previously and successfully isolated and expanded* in vitro* human adipose-derived stem cells (ADSCs) and observed their regenerative potential [22–24]. Based on current knowledge about the expression profile of inflammatory proteins, we aimed at analyzing the capacity of allogenic equine MSCs to integrate and influence the expression of these proteins in mares' endometrial tissue, possibly obtaining interesting data for translational purposes.

## 2. Materials and Methods

### 2.1. Samples Collection

Endometrial biopsies (40 × 40 × 3 mm) included in this study were collected postmortem from 12 mares: 6 younger than 6 years old and 6 older than 15 years old. Animals were classified according to Kenney's histological classes between healthy (classes I and IIA; *n* = 6) and pathological (class IIB and greater; *n* = 6) mares [25].

### 2.2. Equine ADSCs Isolation

Equine ADSCs were isolated from the fat of mares aging less than 5 years. The region above the dorsal gluteal muscle, at the base of the tail, was chosen as the adipose tissue collection site because of the availability of material, the absence of large veins, and ease of access. Adipose tissue samples (approximatively 100 gr each) were preserved in a sterile phosphate buffered saline (PBS) (Lonza, Walkersville, MD, USA) solution enriched with 1% penicillin/streptomycin (P/S) (EuroClone, Milan, Italy) and 1% amphotericin B (EuroClone) until processing. The samples were digested using a solution of 0.075% collagenase from* Clostridium histolyticum* type II (Sigma-Aldrich, St. Louis, MO, USA) in Hank's Balanced Salt Solution (HBSS) (Lonza), for 3 h at room temperature and in slow agitation. At the end of the digestion, the collagenase activity was blocked with an equal volume of complete Dulbecco's modified Eagle's medium (DMEM) (completed: cDMEM). cDMEM consisted of Dulbecco's modified Eagle's medium (DMEM, Lonza) supplemented with 10% fetal bovine serum (FBS, Bidachem S.p.A., Milano, Italy) and 1% P/S. After centrifugation for 7 min at 500 g, the pellet was washed in PBS and filtered with a 70 *μ*M cell strainer (BD Biosciences, Mississauga, Ontario, Canada). The cell suspension was resuspended in cDMEM, transferred to a 25 cm^2^ tissue culture flask, and then incubated at 37°C and 5% CO_2_ for 15 days. Culture medium was changed every 2 days.

### 2.3. Equine ADSCs Labeling with Quantum Dots

Equine ADSCs at p3 were labeled with Qtracker 705 Cell Labeling Kit (Life Technologies, Carlsbad, CA, USA) following the manufacture's protocol. Quantum dots are nanocrystals made of semiconducting materials that, once inside the cells, provide stable fluorescence that can be traced through several generations and are not transferred to adjacent cells in a population [26].

### 2.4. Equine ADSCs Seeding on Endometrial Tissues

Endometrial biopsies from healthy and pathological mares were cut into 10 × 10 × 3 mm pieces. 5 × 10^5^ equine ADSCs were then seeded onto each piece in cDMEM and incubated at 37°C and 5% CO_2_ for 3 days.

### 2.5. Histological Analyses

Endometrial biopsies were frozen in isopentane and liquid nitrogen. Samples were cut with cryostat (Leica CM1950, Leica Biosystems, Nußloch, Germany) in slivers of 7–10 *μ*m.

Healthy and pathological endometrial tissues before equine ADSCs seeding were stained with hematoxylin and eosin. On the contrary, endometrial samples treated with labeled equine ADSCs were stained with Hoechst 33342. All reagents were purchased from Sigma-Aldrich (Saint Louis, MO, USA).

### 2.6. Real-Time PCR

Total RNA was extracted using the TRIzol Reagent (Life Technologies) from healthy and pathological endometrial tissues before and after ADSCs seeding. The samples were quantified using the NanoDrop spectrophotometer (NanoDrop 1000, Thermo Scientific). For the first-strand cDNA synthesis, 1000 ng of total RNA was reverse transcribed using M-MLV RT (Moloney Murine Leukemia Virus Reverse Transcriptase, Life Technologies) according to the manufacturer's protocol. Equine primers were selected for each target gene with Primer 3 software (Table 1). Real-time PCRs were carried out using the designed primers at a concentration of 300 nM and FastStart SYBR Green Master (Roche Diagnostics, Mannheim, Germany) on a Rotor-Gene 3000 (Corbett Research, Sydney, Australia). Thermal cycling conditions were as follows: 15 min denaturation at 95°C, followed by 40 cycles of 15 s denaturation at 95°C, annealing for 30 s at 60°C, and 20 s elongation at 72°C. Values were normalized to the expression of the *β*-actin internal reference, whose abundance did not change under our experimental conditions. Experiments were performed with 3 different cell preparations and repeated at least 3 times.

### 2.7. Statistical Analysis

Data are presented as mean ± SD and MedCalc 9.0 software was used. The effect of ADSCs therapy on the total population was evaluated using paired matched Wilcoxon's rank test. A *P* value < 0.05 for the analysis was considered significant.

## 3. Results and Discussion

### 3.1. Equine ADSCs Isolation and Expansion

**Figure 1:**
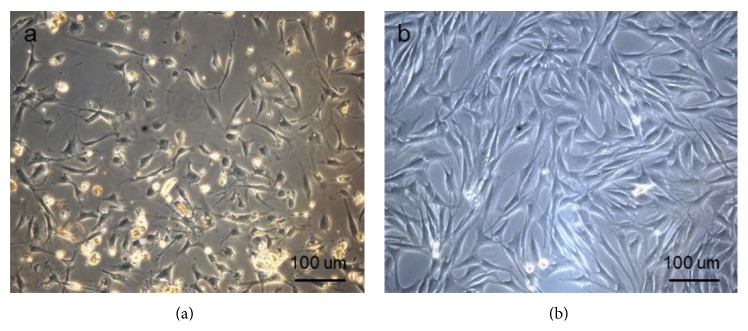
Equine ADSCs at phase-contrast microscope. (a) Equine ADSCs at p0. (b) Equine ADSCs at p3.

Adipose-derived stem cells (ADSCs) were isolated after enzymatic digestion from equine abdominal fat and plated on a 25 cm^2^ tissue culture flask. After 3 days, the nonadherent cells were removed by replacing the medium with fresh complete medium. The adherent cells are defined as passage 0 (p0) cells. At this stage, the cell morphology varies between cuboidal shape and spindle shape (Figure 1(a)). After reaching 80% confluence, ADSCs were trypsinized and expanded until p3.  Figure 1(b) shows that the cells acquire a spindle-shaped fibroblast-like morphology.

### 3.2. Equine ADSCs Labeling and Seeding on Endometrial Tissues

**Figure 2:**
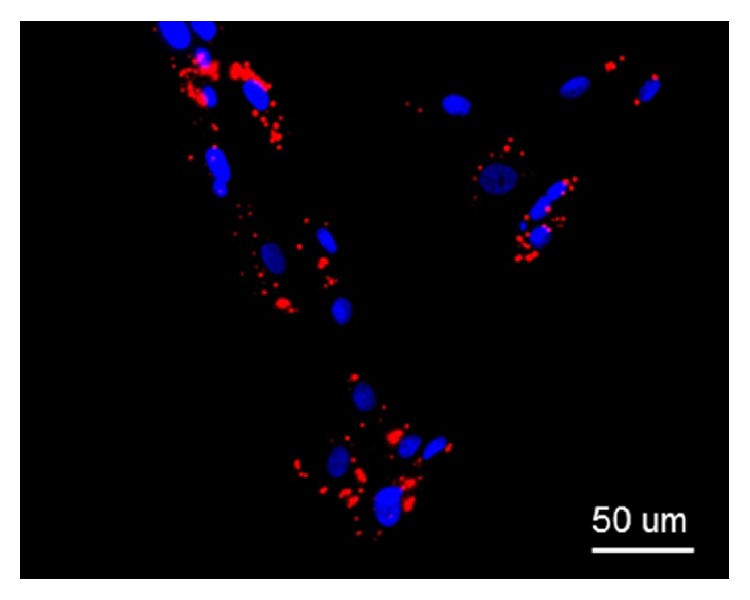
Equine ADSCs at fluorescence microscope. Equine ADSCs at p3 stained with quantum dots (red) and Hoechst 33342 (blue).

In order to verify if equine ADSCs could be able to infiltrate the endometrial tissue, we treated* in vitro* endometrial biopsies with ADSCs previously labeled with quantum dots. Equine ADSCs labeled at p3 show a well distribution of quantum dots nanocrystals in vesicles in the cytoplasm (Figure 2), thus suggesting the efficacy of the method for tracking the ADSCs seeded on the endometrial tissues.

**Figure 3:**
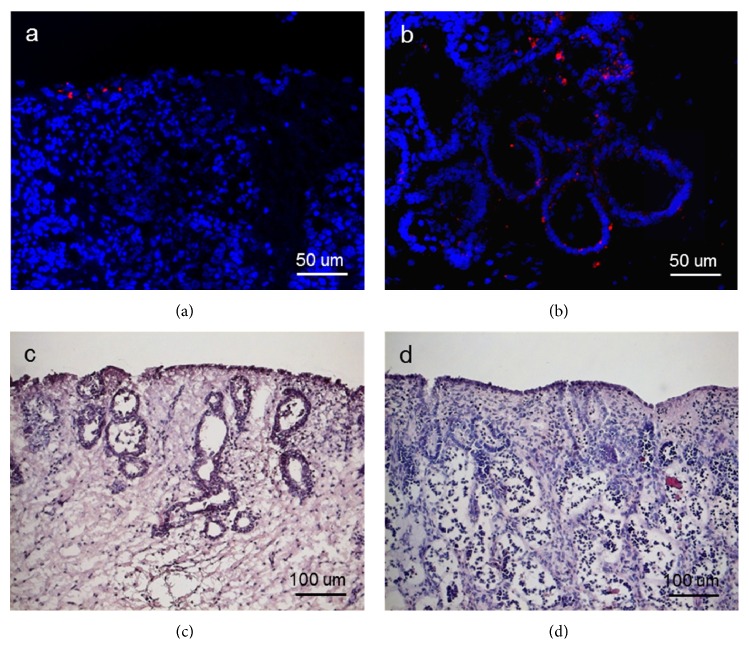
Equine endometrial biopsy from (a, c) healthy and (b, d) pathological mares incubated 3 days with equine ADSCs. (a, b) Fluorescent imaging of equine ADSCs labeled with quantum dots (red); nuclei stained with Hoechst 33342 (blue). (c, d) Hematoxylin and eosin staining of ADSCs-seeded healthy and pathological endometrial tissues.

The presence of fluorescent labeled ADSCs cultivated on endometrial tissue from both healthy and pathological mares has been observed after 3 days from seeding. As reported in Figures 3(a) and 3(b), the equine ADSCs were detected in both of the tissues, although in major quantity in the pathological samples. Furthermore, in the endometriosis-affected tissues, the labeled ADSCs were infiltrated in the periglandular space as well as in single glands. Hematoxylin and eosin staining of the endometrial tissues before ADSCs seeding confirms the different morphological structure between the healthy and the pathological tissues (Figures 3(c) and 3(d)), along with an evident inflammatory condition in the latter.

### 3.3. Gene Expression of Inflammatory Factors before and after ADSCs Seeding

Endometriosis in mares is a degenerative process characterized by epithelial and stromal alterations and fibrosis. Although the disease is well-known and causes severe consequences such as early embryo death, contradictory hypotheses have been made around its etiology, the more supported being a malfunctioning of the immune system and a dysregulation of the inflammatory processes [16].

**Figure 4:**
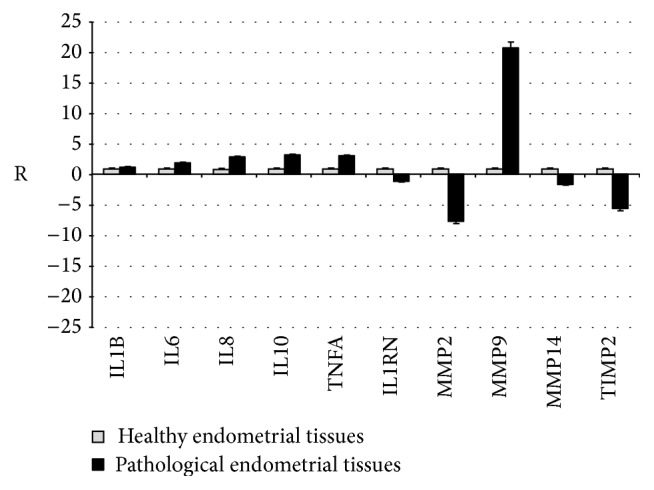
Gene expression levels (R) of cytokines and MMPs in pathological endometrial tissues (black) compared to healthy endometrial tissues (grey) before ADSCs seeding.

In order to test this hypothesis, the expression of different cytokines and MMPs has been evaluated in pathological in comparison to healthy endometrial tissues (Figure 4). Basal expression of interleukin 1 beta (IL1B), interleukin 6 (IL6), interleukin 8 (IL8), interleukin 10 (IL10), tumor necrosis factor alpha (TNFA), and MMP9 was strong in pathologic samples compared to healthy endometrial tissue and particularly significant for IL10 (*P* = 0.02) and TNFA (*P* = 0.003). These results confirmed the observations made by Fumuso et al. [27, 28] and Feghali and Wright [29] regarding a higher expression of IL1B, IL8, and TNFA in endometriosis. Conversely, the higher expression of IL6 and IL10 in pathologic samples contrasted with previous observations, where a low level of IL6 was thought to be associated with an increased risk of the development of endometriosis and high levels of IL10 were auspicated in consideration of its anti-inflammatory properties [27, 28]. Interleukin 1 receptor antagonist (IL1RN) expression levels were lower in pathologic endometrium compared to healthy tissue, confirming its role as a protective factor [17, 30]. MMPs expression levels gave contradictory results, as MMP2 and MMP14 were detected at lower levels in pathologic endometrium, whereas MMP9 was much higher when compared to healthy tissue values. TIMP2 expression followed the MMP2 and MMP14 trend. These discrepancies suggest that a profound dysregulation is active in endometriosis, leading to ECM accumulation and glandular fibrosis [12].

**Figure 5:**
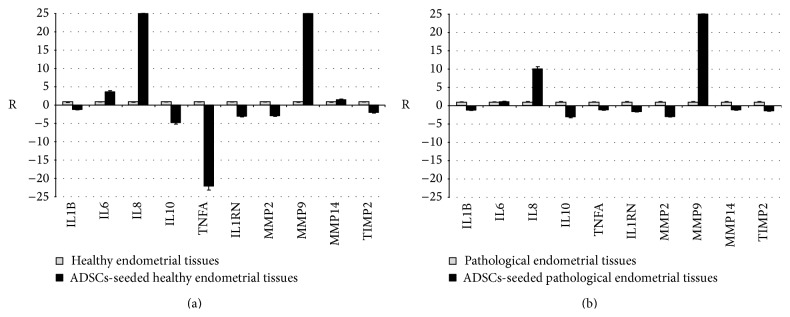
Gene expression profile of cytokines and MMPs after seeding of equine ADSCs in (a) healthy endometrial tissues and (b) pathological endometrial tissues. The results are expressed as ratio (R) with respect to mRNA expression of endometrial tissue without ADSCs.

In order to evaluate the effects of ADSCs seeding on endometrial tissue of healthy and pathological mares, the expression pattern of the same panel of inflammatory secretory factors has been analyzed (Figures 5(a) and 5(b)). The profile obtained from equine endometrial tissues after the treatment with allogenic ADSCs showed a general reduction for expression levels of IL1B, IL10, TNFA, and IL1RN and an increase of expression levels of IL6 and IL8 in both groups. These results suggest both positive and negative effects of equine ADSCs on the inflammatory processes regulation in the endometrium, as a reduction in IL1B and TNFA is auspicious to downregulate chronic inflammatory processes [29], whereas high levels of IL10 and IL1RN are desirable in consideration of their anti-inflammatory properties [17, 28]. A recent study showed contrasting effects for IL6 in the inflammatory process, being related to the recruitment of neutrophils in the early inflammatory phase while inducing neutrophils apoptosis and monocyte phagocytic properties in the late stages [31]. Another cytokine which showed an increased expression rate in both groups after ADSCs seeding was IL8. In consideration of its proinflammatory activity, the effect could be potentially negative in the treatment of endometriosis.

Regarding MMPs and their inhibitors, the expression of MMP2 and TIMP2 decreased, whereas expression levels of MMP9 increased in both pathological and healthy endometrial tissues after ADSCs seeding. As far as MMPs are concerned, the reduced expression of MMP2 after ADSCs culture should have a positive effect in treating endometriosis [12, 19, 21]. The positive effect comprises also the increased expression of MMP9 in both groups. In fact, previous observations made by Aresu et al. [12] detected a standard expression of MMP9 in endometriosis, hypothesizing a positive role coming from an increased activity of tissue remodeling [21]. However we must notice that our pathologic samples already showed a higher expression of MMP9 compared to healthy ones. MMP14 expression levels increased only in healthy tissues. MMP14 can degrade different matrix components and activate other MMPs, in particular MMP2 and MMP9 [12]. Furthermore, TIMPs can influence all MMPs-related processes. Little is published regarding TIMP2 expression in mares' endometriosis [12], but studies on human hepatic fibrosis proved that the recovery phase is characterized by a decrease of TIMP2 expression levels [21].

## 4. Conclusions

All things considered, in the present work we could not detect a clear potential clinical effect of ADSCs on equine endometriosis. The balance between pro- and anti-inflammatory, lytic, and fibrotic environment is very subtle, and many actors are involved in the scene, providing a complex pattern of interactions to consider when trying to influence biologic processes. Nevertheless, we highlighted important differences in the biologic response of endometrial tissue to the exposure to ADSCs, challenging researchers to progress forward with future studies hopefully for the development of a biological therapy in clinical trials.

## Figures and Tables

**Table 1:** Equine primer sequences.

Gene symbol	Forward primer (5′→3′)	Reverse primer (5′→3′)	Product length (bp)
ACTB	CTCCCAGCACGATGAAGA	GTACTCCTGCTTGCTGATCC	125
IL10	GGCACCCAGTCTGAGAACA	GCTGGTCCTTCATTTGAAAGAAAGTC	115
IL1RN	TGTGTCAAGTCTGGTGATGAGA	TGTTTGAGCGGATGAAGGTGA	108
IL1B	CAACGGAGAGAATACAAACCAACAAG	GGCTTCCCATCTTTCATCCCA	141
IL6	AGAACACAACAACTCACCTCATCC	AGAGGAAGGAATGCCCATGAAC	125
IL8	TCCAAGAATTCCTCAGTAAAGATGCC	CCTAGATACTGCGTGGGACAATG	173
MMP14	CAAGATGCCTCCTCAACCCA	GCCACGGTGTCAAAGTTCC	107
MMP2	TGGTCCGTGTGAAGTATGGC	TCGAAGTTGTAGGTGGTGGA	139
MMP9	ACAGTGCCTTTGGGTCCAG	GTACCTCCCGTCCTTGAAGA	102
TIMP2	GCCAAAGCGGTCAGTGAGA	GTAGATGAACTCGATGTCCTTGTCA	132
TNFA	ACGGTGCTTGTGCCTCA	CGGTAACTGCTCTTCCCTCTG	112

ACTB: actin beta; IL10: interleukin 10; IL1RN: interleukin 1 receptor antagonist; IL1B: interleukin 1 beta; IL6: interleukin 6; IL8: interleukin 8; MMP14: matrix metallopeptidase 14; MMP2: matrix metallopeptidase 2; MMP9: matrix metallopeptidase 9; TIMP2: TIMP metallopeptidase inhibitor 2; TNFA: tumor necrosis factor alpha.
